# Awareness and barriers of sacral neuromodulation in women with overactive bladder

**DOI:** 10.1007/s00404-024-07664-2

**Published:** 2024-07-31

**Authors:** Christl Reisenauer, Jürgen Andress

**Affiliations:** Department of Gynecology and Obstetrics, University Hospital Tübingen, Calwerstrasse 7, 72076 Tübingen, Germany

## Introduction

According to national and international guidelines sacral neuromodulation (SNM) is an established, minimally invasive treatment modality for refractory pelvic floor disorders such as overactive bladder (OAB), non-obstructive urinary retention and faecal incontinence. Reported efficacy rates vary between 67 and 85% [1]. It is aimed at providing continuous electrical nerve stimulation of the sacral nerve roots using implanted electrodes and a pulse generator. The procedure involves placement of electrodes into the S3 or S4 sacral foramen during the temporary test phase followed by a permanent pacemaker if the test stimulation is successful. Reorganization of spinal reflexes and regulation of cortical activity are postulated to be the mechanisms of action.

In this article, we will focus on OAB as a common pelvic floor disorder. According to the International Continence Society, OAB is characterized by urinary urgency, with or without urge incontinence, usually with frequency and nocturia, in the absence of causative infection or pathological conditions [2]. Residual urine should be excluded before starting the OAB therapy.

Since the progression to advanced therapies for OAB such as SNM or Onabotulinumtoxin A (BTX) is less than 5% and compliance to medical treatment is poor it has been concluded that more patients could benefit from these second-line therapies (often also referred to as third-line therapies) if conservative treatments are broken down into behavioural therapies and medical treatment) [3].

In order to improve the quality of life of those affected, healthcare providers, healthcare systems and, last but not least, patients need to be aware of SNM. Therefore, we would like to raise awareness and show ways to overcome the barriers for people suffering from pelvic floor disorders.

### General patient and physician barriers

It has been reported that—although OAB has a significant impact on patients’ quality of life and daily activities—symptoms are frequently unreported and underdiagnosed due to the patients’ lack of understanding of the disorder, the patient's reluctance to talk about their symptoms and the patient's belief that OAB is a natural part of aging [4, 5].

In order to explore these barriers further Ücer et al. invited 2250 female patients hospitalized for varied complaints in the Aegean region of Turkey to answer the OAB-V8 questionnaire. The questionnaire included questions on evidence of lower urinary tract symptoms (LUTS). Patients with a total OAB-V8 score ≥ 8 were defined as having OAB symptoms. The aim of their study was to determine the proportion of female patients with undetected OAB symptoms who were referred to hospitals for other diseases and to investigate why these patients did not mention their symptoms. The proportion of patients with OAB symptoms in this study was 40.6%. Nearly 57% of the patients with OAB symptoms had not been previously admitted to any hospital for LUTS. The two most common reasons why women with OAB symptoms did not admit themselves to a hospital because of LUTS were as follows: “I did not think I had a disease” and “The symptoms did not bother me,” with a response rate of 74.7% and “I was ashamed to seek treatment”, with a response rate of 14%. Ücer et al. concluded that the high proportion of patients with OAB in this study suggests that the needs of many of them may not have been met by their primary care providers and public awareness programs on OAB may resolve this problem. Further they suggest that information about OAB should be provided to people via the Internet and media by scientific societies and professional associations in order to increase their awareness [6].

In a landmark study, Milsom et al. collected data using a population-based survey of men and women aged ≥ 40 years, selected from the general population in six European countries (France, Germany, Italy, Spain, Sweden and the United Kingdom). The overall prevalence of OAB symptoms (alone or in combination) was 16.6% and increased with advancing age. Overall, 60% of respondents with symptoms had consulted a doctor but only 27% were receiving treatment [7]. These results suggest that the way doctors diagnose and treat this condition can still be significantly improved.

In another landmark study by Irwin et al. symptom bother and health care seeking behaviour among individuals with OAB has been investigated. This was a nested case-controlled analysis of data from the EPIC study, a population-based, cross-sectional survey of adults in five countries (Canada, Germany, Italy, Sweden, and the United Kingdom) (n = 1434). Among cases, 54% reported symptom bother; rates were similar between men (54%) and women (53%). Approximately 52% of the bothered OAB cases initiated a conversion about their urinary symptoms with their health care providers. Cases reporting symptom bother, particularly those with urinary incontinence (UI), were significantly more likely to use coping techniques and seek health care advice. Limiting fluid intake, using absorbent products, using physiotherapy or exercises, and taking nonprescription or prescription medicines were defined as coping techniques. The authors concluded that clinicians should screen for OAB in all cases and assess symptom bother in those affected to guide diagnosis and treatment [8].

Similarly, low rates of care seeking behaviour has been reported by Brown et al. Fewer than 50% of women with UI and 30% of women with accidental bowel leakage (ABL) or faecal incontinence are seeking care. In this publication Brown et al. identified 12 patient barriers to seeking care for ABL: Lack of knowledge about the condition; Lack of knowledge about treatment; Fear of testing/treatment; Normative thinking; Avoidance/denial; Life impact; Embarrassment/shame; Self-blame; Stigma; Isolation; Provider barriers; Access limitations [9].

Another potential barrier may be the availability of useful online information for patients. For this purpose, Hüsch et al. have examined the content on OAB on various digital platforms (Google, Facebook, Instagram, LinkedIn and YouTube). The source with the highest quantity of useful content was YouTube (100%) and Google (100%), whereas LinkedIn included mostly misleading content (73%). Surgical procedures for treating OAB were only described in 32% and 48% of Google and YouTube results, respectively. On Google, SNM and BTX were described in only 26% of the search results. In contrast, alternative medicine was present in 76%. The authors concluded that a large gap in the information on surgical treatments of OAB has been identified [10].

### Barriers in the primary care sector

Apart from patient awareness a basic knowledge of second-line therapies among primary care providers (PCP) is indispensable to well-inform refractory patients to improve their access to specialized care.

In their cross-sectional study Ghijselings et al. got insight in the extent of knowledge about the second-line therapies for pelvic floor disorders (PFD) among PCPs in Flanders, Belgium. Prevalence of PCPs having awareness about SNM, BTX and posterior tibial nerve stimulation (PTNS) was explored. Respectively, 90, 73, and 5% had ever heard about BTX, SNM, and PTNS. However, only 18% of PCPs had a basic knowledge of the SNM procedure and its indications. The authors concluded that the awareness among PCPs regarding therapies for refractory OAB and other PFD should be increased for the optimization of patient care [11].

### Barriers in specialized care

Clinical inertia (CI) is believed to be another major factor that contributes to the inadequate management of chronic conditions such as OAB. CI has been defined as a failure to initiate or intensify therapy when indicated, or a failure to act despite recognition of the problem [12]. This may lead to discordance with guidelines. Contributors to CI are providers’ overestimation of the care they give, providers’ use of ‘soft’ reasons to avoid therapy (e.g. economic or reimbursement issues) and providers’ lack of education, training or organisation for achieving therapeutic goals.

The main factor of such a risk is when clinicians feel overwhelmed and disempowered, due to characteristics of either the patients or the health care system, including contradictions between guidelines and reimbursement policies. Finally, CI can potentially increase the already existing gap between general practice and specialised care, whereas sustained efforts toward more collaborative work and integrated care are called for [13].

As indicated above, a poor adherence to guidelines may also represent another key barrier for the access of specialized treatment. Guidelines for the management of continence and overactive bladder are generally available across Europe. For a majority of countries, these have been adopted by professional societies in either urology or gynaecology for local use. However, the implementation of guidelines is slow and requires much effort [14]. This was demonstrated by Basu et al. who investigated the opinions of members of the British Society of Urogynaecology regarding the recommendations contained in the NICE guideline on the management of female urinary incontinence. Only 56.8% agreed that the guideline reflects their current practice. And in terms of changing their practice to comply fully with the guideline, 53.3% disagreed [15].

Recently, the barriers facing SNM as a treatment for OAB has been investigated in Canada by Gariscsak et al. identified barriers were lack of available expertise, resources and funding, including operating room time, nursing support, technical staff, and follow-up care post-implantation. More than half of surveyed urologists believe SNM is underused [16]. Although the initial cost can be a deterrent, economic models both in Canada and internationally have demonstrated the cost-effectiveness of SNM versus medical treatment for refractory OAB at 5- and 10-year follow-up [17–19].

### The need for patient-centered care and shared decision-making

Davenport et al. explored the decision-making process in women with OAB who do not pursue third-line therapies using a qualitative approach. Insufficient in-office education was thereby the most common modifiable barrier to third-line therapy. Study participants were heavily influenced by outside factors including the opinions of friends and the media. Negative experiences with less-advanced options and treatment fatigue negatively affected participants’ perceptions of third-line therapies. The authors concluded that office education is tremendously important to patients’ understanding of OAB, expectations of therapy, and treatment compliance. Education about third-line therapy counseling should be incorporated into the initial office visit. This may mitigate expectations, improve patient compliance, and promote graduation to advanced therapy in women who later go on to develop refractory symptoms [20].

Similarly, Moskowitz et al. emphasized the educational component of OAB treatment in order to provide patients with optimal care, and yet it is often overlooked due to time constraints and the belief that it will not improve outcomes. It has been reported that the use of third-line therapies for OAB was significantly higher among board certified FPMRS physicians (Female Pelvic Medicine and Reconstructive Surgery) at a tertiary referral center. Of all patients seen for OAB at their institution 3.5% received third-line therapy compared with 14.1% of those seen by FPMRS providers who are using a care algorithm that educates OAB patients on all of their treatment options from the first visit to illustrate all steps in the treatment algorithm. This combats discouragement if first- or second-line treatments do not provide effective relief of symptoms. Moreover, the authors conclude that their FPMRS providers have a close relationship with primary care providers and in the community, which increases the likelihood of referral to a specialized center early in the OAB treatment course [3].

Since advanced therapies for OAB such as SNM and BTX have different benefit–risk profiles, patient preferences will also play a key role in the process of shared decision making. In a pioneering study with 50 women with OAB by Balchandra et al. 74% preferred BTX and 26% preferred SNM. The Botox group seemed more likely to need quicker results with easy access to the treatment modality, whilst the SNM group seemed keener to focus on a more permanent option with a known interval for the repeat procedure [21]. In contrast, SNM was greatly preferred over BTX in other patient preference studies [22, 23]. These different findings might be explained by the disparity between patient and physician preferences which may impose inadvertently a bias in these surveys [24].

Apart from studies, it make sense to switch from BTX to SNM and vice versa if the OAB therapy does not work/ no longer works.

### MRI as a barrier?

Magnetic Resonance Imaging (MRI) is increasingly a fundamental component of the diagnostic pathway across a range of conditions. It has been estimated that at least half of patients with pacemakers or neurostimulators will have a clinical indication for an MRI examination over their lifetime [25].

Until the beginning of 2020 standard recharge-free SNM devices were not approved for full-body MRI scans, so that SNM was considered as a contraindication for patients with a regular need for MRI examinations such as patients with underlying neurological diseases (e.g. multiple sclerosis). With new SNM technologies (Medtronic Interstim SureScan, Minneapolis, MN and Axonics r-SNM, Irvine, CA) that barrier has been overcome. Specifically, all Axonics SNM systems are labelled “MRI conditional” (1.5 and 3 T) as well as the Medtronic SureScan systems (tined lead 978B1 in combination with Interstim II or Interstim X), whereas the legacy Medtronic devices (tined lead 3889, 3093) are not approved for full-body MRI examinations. Rechargeable devices from both manufacturers are also MRI compatible (1.5 and 3T), however, the exact conditions for MRI examinations may differ such as effective RF magnetic field, specific absorption rates, gradient magnetic field or the need for prior impedance checks. For example, in a recent study by Perrouin-Verbe et al. 25% of enrolled OAB patients had already undergone an MRI examination within a 24-month period [26]. However, barriers in daily practice still exist in some countries when patients with pacemakers or neurostimulators are referred to radiologists for MRI examinations, even if the system is MRI compatible. A recent survey of MRI departments in England showed only 53% of units will scan patients with “MR Conditional” cardiac implantable electronic devices (CIED), and there remains an estimated tenfold service underprovision [27, 28]. Moreover, barriers exist at multiple levels from referrer to reporting radiologist. Patients with CIEDs are approximately 50 times less likely to be referred for MRI than the general population. Therefore, workflows need to be adjusted in order to incorporate time and collaboration from multiple hospital departments [27, 29–31].

### Time for a political campaign

On 8 November 2023, the European Association Urology (EAU) joined 22 scientific, professional, patient and non-profit organizations in launching a manifesto for policy reform on Continence Health in Europe. The manifesto was launched at the first Continence Health Summit in Brussels and aims to raise awareness among EU and national policymakers of continence health problems and to instill the need for action. The summit presents the results of a report on the socio-economic and environmental costs of continence health problems. This research on the economic burden of urinary incontinence reveals that the cost of continence care will reach an estimated €69.1 billion in Europe in 2023. If no action is taken to support continence health, the economic burden could rise by 25% in 2030 to €86.7 billion. This financial burden becomes considerably higher when including caregiver costs [32].

## Conclusions

Raising awareness and overcoming the identified barriers in the diagnosis and treatment of pelvic floor disorders, particularly OAB, can make sacral neuromodulation accessible to a larger number of patients. Better care for OAB patients significantly will improve their quality of life and contribute to a long-term reduction of healthcare costs. In addition to scientific societies and professional associations, every urogynecologist can make a valuable contribution to promote sacral neuromodulation in the treatment of the OAB.
